# Posterior Reversible Encephalopathy Syndrome in a Patient with Newly Diagnosed HIV Infection and End Stage Renal Disease

**DOI:** 10.1155/2013/473618

**Published:** 2013-04-30

**Authors:** Mohankumar Kurukumbi, Maria I. Castellanos, Amanda K. Crawford, Shreyas D. Gowdar, Annapurni Jayam-Trouth

**Affiliations:** ^1^Department of Neurology, Howard University Hospital, 2041 Georgia Avenue Northwest, Washington, DC 20001, USA; ^2^Department of Internal Medicine, Howard University Howard, 2041 Georgia Avenue Northwest, Washington, DC 20001, USA

## Abstract

Posterior reversible encephalopathy syndrome (PRES) is a clinicoradiological syndrome in which patients present with an acute or subacute clinical presentation of seizures, visual disturbances, headache, and altered mental status. The pathophysiology of PRES may be explained by endothelial dysfunction that leads to transudation of fluids and protein, resulting in vasogenic cerebral edema. PRES is typically associated with many conditions such as hypertension, uremia, immunosuppressive drugs, and sepsis. This is a case report of a 39-year-old woman with untreated HIV infection and end-stage renal disease (ESRD) who developed PRES with a normal blood pressure and no other known causes of PRES. Untreated HIV is associated with known endothelial dysfunction and we believe that this, in combination with her untreated end-stage renal disease, contributed to her unique presentation of PRES. Although uncommon in HIV-infected patients and challenging to diagnose, prompt recognition of PRES is critical to provide appropriate care and ensure reversibility of the vasogenic edema seen in PRES.

## 1. Introduction

Posterior reversible encephalopathy syndrome (PRES) is a clinicoradiological syndrome originally described in 1996 by Hinchey et al. [1]. Patients with PRES have an acute or subacute clinical presentation of altered mental status, encephalopathy, headache, new onset seizures, and visual disturbances in association with neuroimaging studies consistent with white matter cerebral edema [1]. 

The hallmark of this rare clinical entity is the reversibility once precipitating factors have been controlled.

There have been few case reports in which PRES has developed in HIV-infected patients [2–5], including one case in which HIV itself was a risk factor for the development of the syndrome [6]. Here we report a case of PRES in a patient whose only risk factor for development of PRES is HIV and renal failure. 

## 2. Case Presentation 

A thirty-nine-year-old African American female with no significant past medical history presented to the emergency department cachectic and “feeling sick” for two weeks with generalized fatigue, malaise, and productive cough with hemoptysis. Review of systems was positive for anorexia, unquantified weight loss, nausea, vomiting, and dizziness. She had no history of hypertension, hematologic disorders, or cancers in herself or in her family. 

The clinical examination at the time of admission revealed severe pallor and oral candidiasis with the rest of the examination benign. Her mental status was normal and she was alert and oriented to person, place, and time. Medical research scale (MRC) grade was 4/5 in all extremities, likely secondary to deconditioning and severe anemia. The patient's vital signs were normal, including a blood pressure of 122/65 mm Hg.

On admission, laboratory analysis revealed severe abnormalities including blood urea nitrogen (BUN) of 138 mmol/L, serum creatinine of 22.3 *μ*mol/L, hemoglobin of 4.9 g/dL, and hematocrit of 16.4% with a normocytic anemia. Arterial blood gas revealed pH 7.17, pCO_2_ 18.8 mm Hg, pO_2_ 143 mm Hg, and oxygen saturation of 99 percent on room air. The other laboratory parameters including white blood count, chemistry panel, platelets, and liver function tests were within normal limits. Urine toxicology screen and pregnancy tests were negative.

She was admitted to the intensive care unit and placed on droplet precautions until tuberculosis was ruled out. She was given 4 units of packed red blood cells after ruling out reversible causes of blood loss. Gynecologic history included eleven live births with poor antenatal care. During the hospital stay, the patient was diagnosed, for the first time, with new onset ESRD classified as Stage 5 chronic kidney disease. It had gone unchecked and the patient was unaware of any kidney issues. During this admission, She received multiple rounds of dialysis which brought her blood urea nitrogen and creatinine ratio (BUN/Cr) to near normal ranges over 3 days. Also, due to the patient's oral candidiasis her HIV status was checked. HIV ELISA and Western Blot were positive with a CD4 T lymphocyte count of 112 cells/*μ*L and a viral load of 780,000 cells/mm^3^. 

The patient was stable for the first 4 days until she had 2 seizures within 24 hours and became nonverbal, uncooperative, and encephalopathic. By this point, her BUN/Cr was brought down to 25/6.2 from the rounds of dialysis. Her blood pressure during the period of seizure was 152/96 mm Hg, which was thought to be secondary to the seizures, and electrolytes were within normal limits. She responded to painful stimuli, pupils were equally reactive, deep tendon reflexes intact, MRC rated 3/5, and there was no papilledema or neck stiffness. Keppra was started for seizure control. 

**Figure 1:**
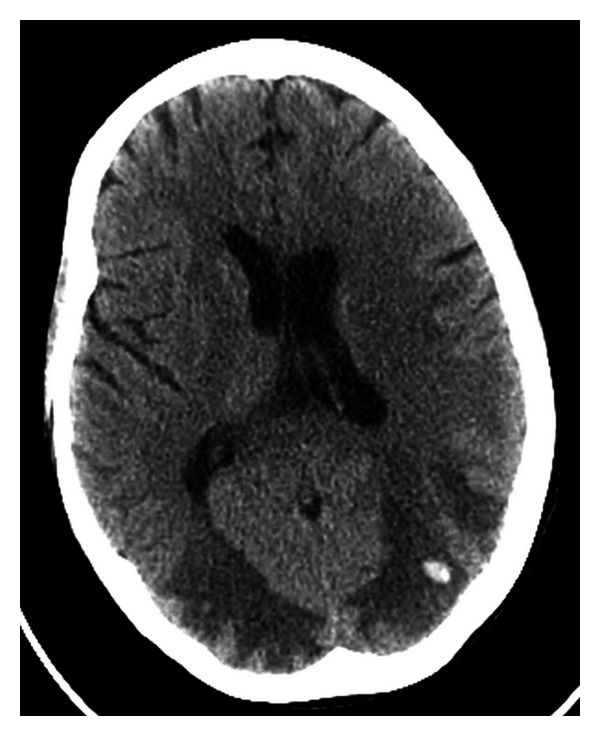
CT without contrast, hypodensity noted in the posterior parietal-occipital regions bilaterally and a small hemorrhage in the left parietooccipital region.

**Figure 2:**
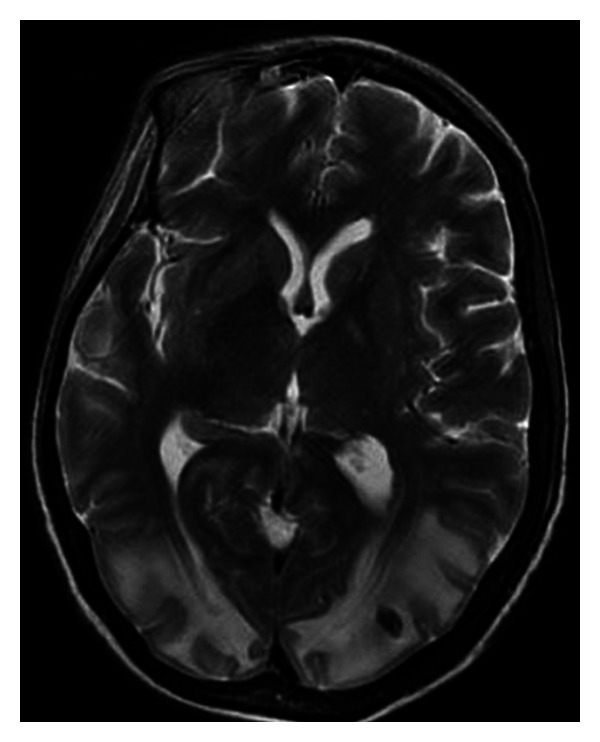
Axial T2-weighted MRI image without contrast demonstrating hyperintensity in the occipital and parietal regions bilaterally, with small foci hemorrhage in the parieto-occipital region.

**Figure 3:**
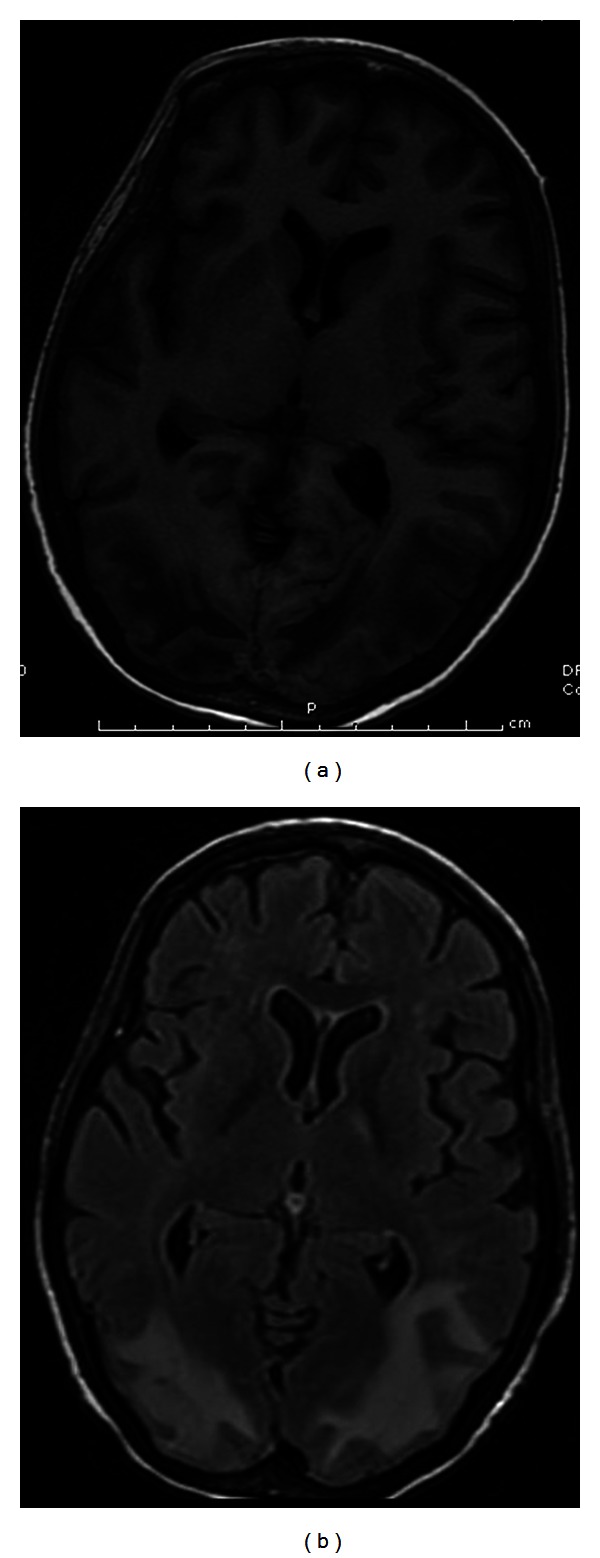
(a) Axial T1-weighted MRI image showing hypointensity in the occipital and parietal regions bilaterally. (b) Axial FLAIR with gadolinium MRI image showing lack of enhancement.

Computed tomography (CT) without contrast showed white matter hypodensity in the posterior parietal-occipital lobes with the remaining findings within normal limits (Figure 1). A CT performed the following day confirmed the presence of hypodense regions but with newly noted small hemorrhages within the parietal and occipital regions. Magnetic resonance imaging (MRI) without contrast revealed areas of T2 prolongation in the subcortical white matter, predominantly in the occipital and parietal regions with scattered foci of susceptibility artifact bilaterally in the occipital lobes. The largest focus measured 10 cm × 0.6 cm in the left occipital lobe (Figure 2). A repeat MRI with gadolinium showed a stable image with no pathologic enhancement and the foci of hemorrhage remained stable (Figure 3).

Given that PRES is a diagnosis of exclusion and based on clinical and radiographic findings, infectious causes of this acute change in mental status were ruled out. Cerebrospinal fluid analysis showed protein of 164 mg/dL, glucose of 49 mg/dL, 630 RBCs/mm^3^, 0 WBCs/mm^3^, and LDH of 37 U/L. No acid fast bacilli were seen. Polyomavirus, herpes simplex virus 1 and 2, cytomegalovirus, Epstein-Barr virus, *Toxoplasma Gondii* IgG, and VDRL were negative. Two sets of blood and urine cultures were negative. There were no further complications with mental status and her cognitive function recovered completely. The patient's vital signs, hemoglobin, and BUN/Cr were stable when she was discharged to home with follow up at the HIV and renal clinics.

## 3. Discussion

Patients with PRES have an acute or subacute clinical presentation of altered mental status, encephalopathy, headache, new onset seizures, and visual disturbances in association with neuroimaging studies consistent with white matter cerebral edema [1]. Findings in MRI imaging reveal hyperintense signaling on T2-weighted and fluid-attenuated inversion recovery (FLAIR) images and hypointense signaling in T1-weighted images, indicative of cerebral edema. The regions most commonly affected are the parietal and occipital lobes bilaterally; although, the frontal lobe, temporal lobe, cerebellum, and brainstem regions have also been reported to be affected [7]. 

Many conditions exist that are commonly associated with PRES, including hypertensive encephalopathy, preeclampsia/eclampsia, immunosuppressive drugs, renal disease, fluid overload, autoimmune diseases, and sepsis [8]. The clinical and neuroimaging findings tend to be reversible once the underlying cause of PRES is identified and treated. Reversibility of the clinical and radiological findings may take between 5 days and 17 months, although there have been reports where normal function is not fully regained [9]. 

The exact pathophysiology of PRES remains unknown. However, three hypotheses exist that may explain the radiological findings in PRES and are related to (1) disorganized cerebrovascular autoregulation, (2) endothelial dysfunction, and (3) vasospasm related to acute increases in blood pressure [1, 6, 10]. Sudden elevation of systemic blood pressure causes cerebrovascular autoregulation failure and breakdown of the blood brain barrier, resulting in hyperperfusion. This loss of autoregulation more prominently affects the parieto-occipital regions because there is decreased sympathetic innervation in the posterior cerebral arterial circulation [11]. The loss of cerebral autoregulation leads to arteriolar vasodilation and endothelial dysfunction, resulting in transudation of fluids and proteins, causing cerebral vasogenic edema [11]. Moreover, cerebral vasospasm can lead to hypoxia and cytotoxic ischemia leading to edema predominantly found in the parietal-occipital regions and watershed areas of the brain [10].

In the setting of renal failure, azotemia may cause interstitial brain edema by increasing the permeability of capillaries and cytotoxic edema by direct injury of the brain parenchyma [12]. The overall occurrence of PRES is currently unknown [9]. However, a retrospective study of 36 patients with PRES reported 45% (17 patients) had the comorbidity of renal failure [7], making renal failure a common condition found in patients diagnosed with PRES. There is one case report of recurrent PRES in a patient with HIV on dialysis [13]. PRES can be a complication of dialysis-dependent patients, but it is due to uremia that dialysis-dependent patients develop as opposed to dialysis disequilibrium syndrome that occurs within hours of treatment [14]. 

There are several case reports that describe PRES occurring in patients infected with HIV [2–5]. Three case reports are in the setting of hypertensive crisis [2, 3], one of which was indinavir induced [3]. Other reports have been in association with hypercalcemia and disseminated coinfection with tuberculosis that developed PRES after being started on anti-TB and ART therapy [4, 5]. However, none of these reports document HIV alone being a risk factor for PRES. Nightingale et al. reported a case in which HIV alone may constitute a risk factor for PRES [6]. 

HIV has been shown to be an independent risk factor for endothelial dysfunction and vascular disease [15]. HIV is known to induce endothelial dysfunction and apoptosis; HIV may infect endothelial cells and induce endothelial inflammation as noted by increased levels of circulating endothelial inflammatory markers [16]. Furthermore, blood-brain barrier dysfunction is commonly seen in HIV-infected individuals and HIV is known to increase the permeability of the endothelium [15]. 

In our patient, who presented with renal failure due to HIV nephropathy, the development of altered sensorium and seizures occurred on day 4 of hospitalization. Of note, our patient did not have a severe elevation of blood pressure and her BUN/creatinine ratio was 25/6.2. PRES can be seen in the absence of hypertension in 20%–40% of cases of patients [9]. Our patient did not present with any other associated etiologies known to cause PRES. Therefore, it is within reason to infer that our patient's severely immunocompromised state and coexisting renal failure contributed significantly to endothelial dysfunction and resulted in the development of PRES.

Other rare contributing factors were considered in our patient. PRES has been observed as a very rare neurological complication of blood transfusions. In these case reports, the patients had a history of severe chronic anemia and were transfused with a high volume of blood in a short period of time [17]. The hypothesis that may explain this phenomenon is related to volume overload that results in the cerebral dysautoregulation due to a rapid increase in hemoglobin [17]. Our patient received 4 units of blood four days prior to the onset of seizures and altered sensorium. We believe that the blood transfusions she received did not contribute to the development of PRES as she received blood products over a gradual period of time.

## 4. Conclusion

The underlying mechanism of PRES, in our patient, could be due to endothelial dysfunction caused by uncontrolled, undiagnosed, and untreated HIV infection. Along with HIV, renal failure also contributed to endothelial dysfunction. To our knowledge, this is only the second case of PRES seen in an HIV-infected patient without concomitant hypertension. PRES should be considered in patients who are normotensive and HIV should be more widely recognized as a risk factor for the development of PRES. The presence of HIV makes the diagnosis of PRES challenging as other conditions may mimic similar clinicoradiological presentations and prompt recognition of this condition is critical to ensure reversibility of the vasogenic edema seen in PRES.

## Abbreviations

PRES: Posterior reversible encephalopathy syndrome
HIV: Human immunodeficiency virus
ESRD: End-stage renal disease
CT: Computed tomography
MRI: Magnetic resonance imaging
BUN: Blood urea nitrogen
Cr: Creatinine
TB: Tuberculosis
ART: Antiretroviral therapy
FLAIR: Fluid-attenuated inversion recovery
ELISA: Enzyme linked immunosorbent assay
MRC: Medical research scale
IgG: Immunoglobulin G
LDH: Lactate dehydrogenase
VDRL: Venereal disease research laboratory
RBC: Red blood cell
WBC: White blood cell.
